# Optimal Scale-Invariant Wavelet Representation and Filtering of Human Otoacoustic Emissions

**DOI:** 10.1007/s10162-024-00943-4

**Published:** 2024-05-24

**Authors:** Arturo Moleti

**Affiliations:** Department of Physics and NAST Centre – University of Rome ‘Tor Vergata’, Rome, Italy

**Keywords:** Otoacoustic emissions, Scale-invariance, Wavelet transform, Time-frequency analysis, Cochlear modeling

## Abstract

Otoacoustic emissions (OAEs) are generated in the cochlea and recorded in the ear canal either as a time domain waveform or as a collection of complex responses to tones in the frequency domain (Probst et al. J Account Soc Am 89:2027–2067, 1991). They are typically represented either in their original acquisition domain or in its Fourier-conjugated domain. Round-trip excursions to the conjugated domain are often used to perform filtering operations in the computationally simplest way, exploiting the convolution theorem. OAE signals consist of the superposition of backward waves generated in different cochlear regions by different generation mechanisms, over a wide frequency range. The cochlear scaling symmetry (cochlear physics is the same at all frequency scales), which approximately holds in the human cochlea, leaves its fingerprints in the mathematical properties of OAE signals. According to a generally accepted taxonomy (Sher and Guinan Jr, J Acoust Soc Am 105:782–798, 1999), OAEs are generated either by wave-fixed sources, moving with frequency according with the cochlear scaling (as in nonlinear distortion) or by place-fixed sources (as in coherent reflection by roughness). If scaling symmetry holds, the two generation mechanisms yield OAEs with different phase gradient delay: almost null for wave-fixed sources, and long (and scaling as 1/*f*) for place-fixed sources. Thus, the most effective representation of OAE signals is often that respecting the cochlear scale-invariance, such as the time-frequency domain representation provided by the wavelet transform. In the time-frequency domain, the elaborate spectra or waveforms yielded by the superposition of OAE components from different generation mechanisms assume a much clearer 2-D pattern, with each component localized in a specific and predictable region. The wavelet representation of OAE signals is optimal both for visualization purposes and for designing filters that effectively separate different OAE components, improving both the specificity and the sensitivity of OAE-based applications. Indeed, different OAE components have different physiological meanings, and filtering dramatically improves the signal-to-noise ratio.

## Introduction

The scale-invariance of the cochlea, which approximately holds over a wide frequency range, with the notable exception of a wide apical region, suggests that the optimal representation and analysis of cochlear signals may be obtained using scaling-symmetric time-frequency analysis tools, such as the wavelet transform. In this study, we will show how this technique is best suited for (but not restricted to) the analysis of human otoacoustic emissions (OAE), due to the particularly long phase-gradient (or group) delays (or narrow bandwidths) that are characteristic of the human OAE response [3, 4]. The role of advanced signal analysis and acquisition techniques in the study of a complex system such as the human cochlea should not be underestimated. The main mathematical details of the cochlear response and of the OAE generation are reasonably well predicted by different families of cochlear models. All theoretical models are optimized by comparison of their predictions to experimental observations, which include (but are not limited to) the direct measure of the vibration of the basilar membrane (BM) and other elements of the Organ of Corti (OoC), auditory nerve responses, cochlear microphonics, and OAEs. Dedicated advanced signal acquisition and analysis tools exploit the theoretical knowledge about the mathematical properties of the signal sources to get unambiguous and detailed information about the system under investigation and provide the effective representation and filtering of the experimental signals that are essential to establish a reliable link between theory and experimental data.

Although the cochlear response is strongly nonlinear, analysis tools and concepts, designed and valid for linear systems only, may be applied, to some extent, to the study of the cochlear response, including the otoacoustic emission (OAE) response. For example, the impulse response, or its conjugated frequency response in the Fourier formulation, and the transfer function, with its poles and zeros, in the Laplace formulation, whose universal meaning is limited to linear systems, can be used to describe, to some extent, the response of a nonlinear system in a given stimulus level range. Although the equivalent-nonlinearity (EQ-NL) theorem has been demonstrated by de Boer [5] only for the cross-correlated output of a system under random noise stimulation, it is often invoked outside its limits of validity to suggest that the response of a nonlinear system to a given stimulus level can be represented as that of an equivalent linear system. In any case, it is not forbidden to test a nonlinear system using the typical experimental tools used for linear systems. The experimental response to a delta-like stimulus of a given amplitude can still be named “impulse response”, and its Fourier transform (FT), or a collection of responses to sinusoidal stimuli of the same amplitude and initial phase, can still be (inaccurately) named “frequency response,” although they do not have the predictive power of the linear case about the response of the nonlinear system at a different stimulus level, and/or for stimuli of different spectral distribution. Experimentally, the local BM response functions to clicks and tones show approximate similarity [6].

In this study, we are focusing on finding the best representation and filtering tools for signals generated by systems showing specific cochlear symmetries. Indeed, a few cochlear features deserve attention, because they impact on the analytical form of the signals generated by the cochlea itself, and, among them, of the OAEs. One of these features is causality, which implies a specific relation between the real and imaginary parts of the frequency response [7, 8], another one is the zero-crossing intensity invariance of the impulse response [9, 10]. Such features impose important constraints to the analytical form of theoretical models, and they may also help designing signal filtering techniques that reject as artifacts the signal components that do not respect these constraints [11].

### Scaling Symmetry

This study is focused on signal analysis techniques that optimally exploit scale-invariance, a property of several physical systems, which approximately holds in the cochlea, independently of most details of the specific cochlear models. It means that the physics of the cochlear response to sound of a given frequency is the same at all frequency scales [12–14], provided one moves to the corresponding spatial place along the cochlear longitudinal direction. The approximate scale-invariance of the auditory nerve fiber and OAE tuning curves over a wide basal frequency range [15, 16] is the experimental evidence supporting the hypothesis that scaling symmetry holds in the basal part of the cochlea. If the suppression tuning curves are plotted against scaled frequency (ratio between suppressor frequency and probe frequency), they superimpose to each other. In a scaling cochlea, a given longitudinal displacement is associated with the same frequency ratio, or relative bandwidth, at all frequency scales, as in a piano keyboard. The number of wavelengths of the traveling wave from the base to the resonant place of a given frequency is independent of frequency, as well as the phase at the peak of the response. This invariance is not limited to the resonant place, for example, the phase of a traveling wave (TW) component of frequency *f*_0_ at a place *x*(*f*_1_), resonant at frequency *f*_1_ = *cf*_0_, is independent of *f*_0_, as long as *c* is a constant. The duality of the cochlear response is a remarkable consequence of scale-invariance [17]. It is equivalent to measure the BM response at a given cochlear place *x*_0_ as a function of frequency *f*, or at a given frequency *f*_0_ as a function of the position *x*. This is a relevant fact, because one experiment (the first one) is much simpler and less invasive than the other. Indeed, spanning a wide stimulus frequency range in optical measurements of the BM response requires just one optical access point along the BM, while measuring the response to a single frequency requires repeating the optical setup procedure at different places. The opposite is generally true for the numerical solutions of a nonlinear cochlear model, because computing the response of the whole cochlea to a sinusoidal stimulus of given frequency requires just one simulation, while exploring the response of a single cochlear place as a function of frequency requires running several simulations using sinusoidal stimuli of different frequencies. On the other hand, one should always consider that, in principle, for a nonlinear system, computing the impulse response of the place as a function of frequency using a wideband (e.g., click) stimulus does not provide the same information for a nonlinear system. Indeed, the experimentally observed similarity between the BM (and OAE) responses to clicks and tones [18, 19] is also related to another property of the cochlear transmission, i.e., the temporal and spatial dispersion of different frequency components of the traveling wave.

In a scale-invariant system, the intrinsic frequency bandwidth $\gamma$, reciprocal of the decay time of the impulse response, is proportional to frequency. In other words, the tuning, or quality factor *Q* of the response, is independent of the frequency scale. This scaling property should be matched by the time-frequency analysis tools used to represent and filter the signals generated by such a system, because otherwise, the time and frequency resolution of the analysis (always reciprocal to each other) would scale with frequency differently than those of the signal components. This is particularly relevant when the system is studied over a wide frequency range (several octaves) and the scaling symmetry approximately holds over such large intervals. In the case of the cochlea, scale-invariance is strictly related to the exponential spatial dispersion of its response to different frequencies (dependent on a single length scale) and to its qualitatively uniform mechanical organization. Indeed, the dependence of the local resonance frequency on the longitudinal coordinate is due to quantitative structural changes (BM width, thickness, cavity tapering, etc.) within a qualitatively uniform structure. In other sensory systems, either artificial or naturally developed by evolution, the response to stimuli of very different frequencies may be performed by subsystems based on different physical mechanisms, but this is not the case of mammalian hearing. This way, the same features that yield high-frequency resonances (e.g., low inertia) also imply fast variations of the waveform envelope, i.e., large bandwidth, and vice-versa.

### Cochlear Scaling Symmetry Breaking: Apical to Basal Transition

The apical region of the cochlea deserves a separate discussion, because a rather sharp scale-invariance breaking, often named apical-basal transition [20–23], occurs at about 1–2 kHz in humans. In other mammals, the same transition occurs at higher frequencies [15]. This “discontinuity” manifests itself in several ways, including a departure of the tonotopic map from the logarithmic scale-invariant functional form, and an experimentally observed dependence of cochlear tuning on frequency. Its origin is still controversial; it has been suggested that it could be related to the slow continuous variations of cochlear parameters, such as the tapered scalae height [24], the ratio between the local tonotopic frequency and the local characteristic frequency of the low-pass filter [25] associated with the build-up of the outer hair cell (OHC) transmembrane potential [26, 27], and/or to the frequency dependence of the viscous damping [28, 29]. It is not surprising that such a relatively sharp discontinuity may be caused by a relatively slow longitudinal variation of cochlear parameters. Indeed, some cochlear parameters affect the BM response in a threshold-like nonlinear way: for example, the scalae height is related to the sharpness of the transition between long-wave and short-wave behavior in the peak region and to the intensity of the related fluid-focusing phenomenon [21, 24].

Although most experiments were dedicated, for practical reasons, to the basal part of the cochlea, one should consider that the peculiar behavior of the “apex” is not a negligible fact. Indeed, the apical part extends over roughly one half of the cochlea, with significant variations of this ratio among different mammalian species [30].

### Conformity to Scaling of OAE Generation Mechanisms and Acquisition Paradigms

For the sake of clarity, when discussing otoacoustic experiments, it is necessary to make a distinction between the term “scaling symmetry breaking,” which will be reserved here to the actual breaking of the symmetry of the cochlea, due, e.g., to dependence of tuning on frequency, tapering, apical-basal transition, and the term “conformity to scaling” associated with the OAE generation mechanisms and with the acquisition paradigms. In other words, while the cochlear scaling symmetry may either hold or be broken over a specific frequency range, the OAE generation mechanisms, and the different stimulus paradigms used to evoke different kinds of OAEs, may either conform or not to the same symmetry. A generation mechanism conforms to scaling if the generation place is a function of frequency scaling as the tonotopic map. This is the case of any wave-fixed mechanism, such as nonlinear distortion, whereas place-fixed mechanisms, such as linear coherent reflection, do not conform at all to this rule. Analogously, the fixed-ratio DPOAE stimulus paradigm using a constant frequency ratio between the stimulus tones *f*_2_ and *f*_1_ conforms to scaling, while the fixed-*f*_2_ paradigm, in which only *f*_1_ is varied, does not.

### OAE Generation Mechanisms and Group Delay

A remarkable consequence of scaling symmetry is related to the specific phase-frequency relation that may be theoretically predicted for OAEs generated by different mechanisms [2, 31, 32]. We will show how the scale-invariant wavelet representation and filtering tool is particularly useful for analyzing OAEs, because they are generated by two mechanisms, one (nonlinear distortion) that conforms to scaling symmetry, and another one (coherent reflection by roughness) that does not conform at all to it. Nonlinear distortion is a wave-fixed mechanism, meaning that the generation place is a function of the frequency, and moves, when the stimulus frequency is changed, to the corresponding place according to the tonotopic scaling relation between frequency and position. Linear coherent reflection by roughness is a place-fixed mechanism, meaning that the position of each backscattering place does not change at all with the stimulus frequency. If scaling symmetry holds, and if the considered OAE generation mechanism and the evoking stimulus paradigm both conform to scaling, the phase of the corresponding OAE component is independent of frequency, i.e., its group delay is null, at all frequencies. For a place-fixed generation mechanism, scaling symmetry implies [31] that the OAE group delay is still predictable and proportional to the reciprocal of the frequency. The proportionality numerical coefficient mainly depends, in a model-dependent way, on cochlear tuning, and is therefore a slow function of frequency in the case of slow symmetry breaking associated with the frequency dependence of cochlear tuning. It is noteworthy that the functional form of the frequency dependence of experimentally measured OAE quantities may be theoretically predicted if (a) the cochlear scaling symmetry holds, and (b) either both the involved OAE generation mechanism and the experimental paradigm conform to scaling (e.g., the DPOAE distortion component evoked by a fixed-ratio paradigm), or one of them does not conform to it in a mathematically well-defined way (e.g., the DPOAE reflection component evoked by a fixed-ratio paradigm, or the DPOAE distortion component evoked by a fixed-* f*_2_ paradigm, because in both cases the generation region does not change with frequency).

## Methods

### Time-frequency Representation of the Response of Scaling Symmetric Systems

Among the linear time-frequency analysis tools that represent a signal on the time-frequency plane, the wavelet transform (WT) is characterized by the scaling symmetric generation of its basis functions [33]. Each basis function is indeed a scaled (dilated) and time-shifted version of the mother wavelet *h*_0_(*t*), which is some oscillating function localized both in time and frequency domain, fulfilling a few basic mathematical requests (square integrable, zero mean). If the spectrum of *h*_0_ is centered at the angular frequency $\omega$_0_ ≡ 2$\pi$*f*_0_, each wavelet of the basis has the form:

1 $${h}_{\alpha ,\tau }\left(t\right)={h}_{0}\left(\frac{t-\tau }{\alpha }\right)$$

This wavelet is localized at time $\tau$, while *α*, called scaling parameter, determines both the center frequency of each wavelet $\omega$_α_ = $\omega$_0_/*α*, and its bandwidth: Δ$\omega$_α_ = Δ$\omega$_0_/*α*. The scale-invariant dilation algorithm generating the wavelet basis functions ensures that the relative frequency resolution Δ*f*/*f* of the t-f analysis is constant across the whole frequency range. Therefore, it matches the same property of the resonant modes of a scaling symmetric system and is therefore optimal for studying the response of the scaling cochlea [34, 35]. To better understand this concept of “optimal” resolution, we must consider that any time-frequency transform projects the original signal on a basis of functions. Each basis function is localized both in time and frequency domain within what we may define as the time and frequency uncertainty (Δ*t* and Δ*f*, reciprocal to each other) introduced by the analysis in that frequency range. The intrinsic duration and bandwidth (again, reciprocal to each other) of the real response of a system are combined to those of the analysis to yield the final time and frequency uncertainties. Therefore, matching them over the whole measurement range minimizes the uncertainty introduced by the t-f analysis.

For any given choice of the form of the function *h*_0_, there is still a degree of freedom related to the relative frequency resolution Δ*f*/*f*, which can be changed, while respecting the unescapable uncertainty lower limit on the product Δ*f*Δ*t*. In other words, a global optimal compromise, based on the actual t-f distribution of the response, can be found between the frequency resolution and the time resolution of the joint t-f analysis.

The wavelet transform of a waveform* x*(*t*) is obtained by taking its inner product with all the basis functions of the family, which belong to a two-dimensional space parametrized by *α* and $\tau$:

2 $${WT}_{x}\left(\omega ,\tau \right)=\int x\left(t\right){h}_{\alpha ,\tau }\left(t-\tau \right)dt.$$

In the digital implementation of the continuous wavelet transform (CWT), $\omega$ and $\tau$ are evenly distributed over that space, yielding a redundant family of basis functions. The same operation may be more conveniently performed in the frequency domain as a product of complex functions followed by an inverse Fourier transform (IFT):

3 $${WT}_{x}\left(\omega ,\tau \right)=IFT\left(X\left(\omega \right){H}_{\alpha ,\tau }^{*}\left(\omega \right)\right),$$

where the asterisk denotes complex conjugation. The convolution operation of Eq. (2), or the equivalent complex product of Eq. (3), yields a representation of the original signal in which the “intrinsic” width of the signal components along the time or frequency dimensions are convolved with those of the basis functions used for the analysis. In experimental applications of Fourier analysis performed on a finite time interval *T*, a similar situation applies to the spectral line widths, which depend on both the intrinsic width of the line and on the frequency resolution Δ*f* = 1/*T* of the Fourier analysis. For example, an originally monochromatic (sinusoidal) signal at frequency *f*_0_ is represented as a vertical band in the t-f plane with the frequency width of the basis wavelet of center frequency *f*_0_. In this case, the wavelet representation introduces unnecessary uncertainty. Obviously, for a monochromatic stationary signal, the Fourier representation is the best one. A signal component described by a wave packet centered at a delay $\tau$_0_ and at a frequency *f*_0_, with duration Δ*t* and bandwidth Δ*f* = 1/Δ*t*, is represented as an elliptical spot in the t-f plane. The size of such a spot would be minimized, and its aspect ratio preserved, by choosing a family of basis functions such that the basis function centered at *f*_0_ has the same bandwidth (and, therefore, the same duration) as the signal component. If this match is not achieved, unnecessary large uncertainty is introduced along one of the two dimensions (delay or frequency) with no or limited advantage along the other dimension. One should consider that, as the basis functions of the CWT are not orthogonal to each other, the projection of any signal cannot yield a point-like WT, but will yield an elliptical spot whose time and frequency widths are determined by its intrinsic uncertainties and by the local Δ*t* and Δ*f* of the analysis. For this reason, the exact functional form of the mother wavelet is not such a crucial choice, but the values of Δ*t* and Δ*f* can be optimized.

The scaling symmetry of the basis functions generation procedure, Eq. (1), implies that the time and frequency uncertainties introduced by the analysis match as much as possible the intrinsic characteristic time and frequency widths of the frequency components of the response of a scale-invariant system at all frequency scales. For comparison, consider the short-time Fourier transform (STFT):

4 $${STFT}_{x}\left(\omega ,\tau \right)=\int x(t){g}_{\omega ,\tau }\left(t-\tau \right)dt,$$

with

5 $${g}_{\omega ,\tau }\left(t\right)= {e}^{i\omega t}{e}^{\frac{{\left(t-\tau \right)}^{2}}{2{\sigma }^{2}}}.$$

The time and frequency resolution of the STFT are dependent on the single parameter $\sigma$, proportional to Δ*t*, and inversely proportional to Δ*f*. The *t-f* plane is uniformly tiled by identical rectangular tiles, and the choice of $\sigma$ may just change the aspect ratio (Δ*t*/Δ*f ≈ *$\sigma$^2^) of the tiles. This is a non-optimal choice for scale-invariant physical systems, and also for a much ampler class of systems in which the frequency of the resonant modes is strongly correlated with the inertia of the involved subsystem.

After having performed suitable filtering operations in the time-frequency domain, the filtered wavelet transform *WT*_*x*f_($\omega , \tau$) may be inverted to recover filtered spectra or waveforms in the frequency- or time-domain:

6 $$x_{\text{f}}\left(t\right)=\iint {WT}_{xf}\left(\omega ,\tau \right){h}_{\alpha ,\tau }\left(t-\tau \right)d\tau d\omega .$$

The robustness against noise of the wavelet transform was demonstrated superior [36] to that of other linear and nonlinear t-f analysis techniques, for the specific task of evaluating the group delay, or latency, of the coherent reflection OAE components. An interesting variation of the WT is the S-transform [37], which performs similarly to the WT in the OAE analysis.

The strength of the wavelet representation, its scale-invariance, may also become its weakness, when important components of the response are far from being scale-invariant. This is the case, for example, of the very narrow-band components of the TEOAE response associated with spontaneous emissions, which do not respect the expected scaling relations between frequency, bandwidth, and delay. The wavelet transform is not effective for representing, in the same scalogram, synchronized spontaneous OAEs (SSOAEs) and other TEOAEs, because, if optimized for the scale-invariant “bulk” of the TEOAE response, it badly fails to represent the intrinsic narrow band of the SSOAE component.

To solve this problem, other time-frequency representations may be used for OAEs. In the TEOAE case, the matching pursuit technique uses a dimensionally redundant “dictionary” of basis functions whose waveforms may be asymmetrically shaped (e.g., assuming different onset and decay times) to match as much as possible the typical impulse response of the cochlea [38–40]. This way, the TEOAE response may be matched by a small number of resonant responses of given frequency, amplitude, delay, and duration/bandwidth. Such a technique may be effectively used when the TEOAE response, as it often happens in newborns, is dominated by a small set of narrow-band spectral lines, which do not respect scaling, typically recognizable also as SSOAEs [41, 42]. The possibility of shaping the basis function in the time-frequency domain could also be extended further, including, e.g., the frequency glides observed in the BM and auditory nerve response to clicks [43].

Generally speaking, nonlinear time-frequency analysis techniques, like Wigner-Ville and Choi-Williams [44], may also escape the “uncertainty principle.” This is obtained at the cost of introducing “ghost” features in the t-f plane, due to interference terms, which may be minimized by suitable parameter choices [45]. Recently, nonlinear techniques, like concentration of frequency and time (ConceFT), have been used to analyze TEOAEs in the time-frequency domain [46], proving superior to the linear techniques in the optimal simultaneous representation of all TEOAE components, including SSOAEs, adapting to the absence of scaling. Nonlinear time-frequency techniques, such as optimal shrinkage, may also be useful to optimize signal denoising procedures [47], with denoising performances better than those of a classical Wiener filter. These advanced nonlinear techniques require a high degree of preliminary knowledge of the signal behavior, to avoid the risk of applying them outside their validity range. For example, the ConceFT relies on the assumption that the underlying signal is reasonably well represented by a combination of intrinsic mode functions (IMT) with slowly varying phase and amplitude functions. Such constraints make the use of such approaches for non-specialists less straightforward than that of linear methods.

In the supplementary material, a Matlab discretized implementation of time-frequency representation and filtering based on the WT is reported, using the mother wavelet function proposed by Tognola et al. [35], and a fast computation method in which all time convolutions are replaced by products in the frequency domain.

## Wavelet Representation and Filtering of OAEs

### DPOAE Representation and Filtering

The most studied DPOAE is associated with the cubic intermodulation DP generated at the frequency *f*_DP_ = 2*f*_1_ − *f*_2_ by two nearby frequencies *f*_1_ and *f*_2_. These signals are the linear superposition of two backward signals, the backward wave generated in the “overlap” region by nonlinear distortion of the two primary waves at frequencies* f*_1_ and *f*_2_, and the fraction of the forward intracochlear distortion product (IDP) wave that is amplified and reflected by roughness near the *f*_DP_ resonant place. In the conventional frequency domain representation, the DPOAE response is characterized by oscillations of amplitude and phase measured across a wide frequency range, associated with the linear superposition of these two components of different group delays. These characteristic response patterns are named DPOAE “fine structure” [48, 49]. Although the name suggests the presence of some additional resonant mechanism producing the observed spectral peaks, it was soon recognized that the fine-structure pattern is just due to alternate constructive-destructive interference between two components whose phase difference is a monotonic function of frequency. Indeed, the superposition of two (almost) constant-amplitude spectra with different phase gradient delays yields complex spectra with oscillating amplitude and phase.

The DPOAE fine structure was also considered a promising diagnostic tool, because its presence/absence seemed correlated with the peripheral hearing functionality [50]. Classification of the shape of different fine-structure peak shapes was undertaken, as well as the measurement of characteristic observable parameters, like the amplitude and the pseudo-period of the oscillations [51]. A DPOAE complex spectrum with fine structure is a typical example of a signal that is represented in the non-optimal domain; therefore, its main physical features, represented by a small number of parameters, are hidden, or, better, encoded, in the visualized pattern.

A very similar and familiar example is that of the representation of the superposition of two signals of nearby frequencies *f*_1_ and *f*_2_ in the time and in the frequency domain. In that case, the well-known beating patterns are visible in the time domain, but, if one is interested in the physical mechanisms generating that response, the frequency domain provides the optimal representation, immediately displaying two lines of measurable frequency and bandwidth, two parameters of immediate physical modeling interpretation. In the non-optimal time-domain representation, the time periods corresponding to the carrier frequency (*f*_1_ + *f*_2_)/2 and to the modulation frequency *f*_2_ − *f*_1_ can be directly observed, while the “encoded” *f*_1_ and *f*_2_ must be computed, and from the depth of the modulation, one can compute the encoded amplitude ratio. Choosing the “wrong” representation domain cannot destroy information, as long as linear reversible transformations are used, but the relevant physical information may be made more or less easily accessible and comparable to theoretical predictions. The representation issue is not a trivial one, because most physical phenomena are synthesized by a set of observable quantities that is much smaller than the degrees of freedom of the recorded signal (the time samples of the waveform or the frequency bins of the complex spectrum). This is true both for the experimental data and for the theoretical model simulations. The optimal representation allows an immediate visualization of the values of the relevant observable quantities. Adding noise strengthens this argument, because the patterns in which the physical information is encoded may become more difficult to be recognized in the “wrong” representation domain.

**Fig. 1:**
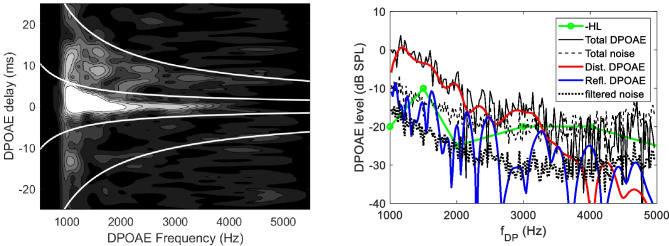
Left: wavelet time-frequency representation of the DPOAE response of a 60-year-old subject. The single-reflection and distortion components are clearly recognizable within the hyperbolic curves, while longer-delay multiple reflections components are visible in the upper part of the plot. Symmetric negative-delay components are also visible in the low-frequency range. A compressively nonlinear intensity map is used to enhance the low amplitude details of the distribution. Right: original DPOAE spectrum and noise and filtered distortion and reflection components. Note the lower noise floor after filtering. The audiometric hearing level (in negative dB HL) is also shown

**Fig. 2:**
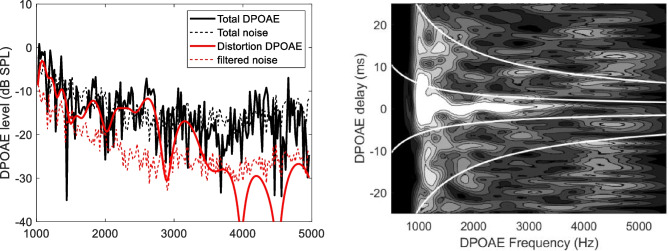
Frequency-domain (left) and wavelet time-frequency representation (right) of a weak DPOAE response. The zero-latency distortion component is clearly visible in the wavelet representation, and in the filtered distortion spectrum (red line), despite the low SNR of the total DPOAE over the whole frequency range

Returning to the DPOAE fine structure, in the frequency-domain representation, the immediately visible modulation amplitude and pseudo-period of the interference pattern, similarly to the beating example, somehow encode the relevant physical information. In the “right” domain, which in this case is the *t-f* domain, the dependence on frequency and phase gradient of the amplitude of each DPOAE component are immediately visualized in well-separated regions of the time-frequency plane, as shown in Fig. 1 (left panel). The complex spectra of the distortion and reflection components may be effectively unmixed by time-frequency domain filtering (right panel). The DPOAE response shown in Fig. 1 was obtained using linear chirp stimuli of amplitude (*L*_1_,* L*_2_) = (61, 55) dB and frequencies *f*_1_*(t)* and* f*_2_*(t)*, with a constant ratio *r* = 1.22, such that the resulting DPOAE response at 2*f*_1_*(t)* − *f*_2_*(t)* swept the frequency range (1–5 kHz) at a constant rate 0.8 kHz/s. The response waveform is decomposed into 50% overlapping windowed frames of 50 ms duration, which are Fourier analyzed to get the amplitude and phase of the current DPOAE frequency component. The speed of the chirp is optimized to match the frequency resolution of the individual spectrum (20 Hz) to the frequency interval between nearby frames. With this Fourier analysis method, either linear or logarithmic chirps may be used, whereas, in the case of the least square fit (LSF) analysis method [52], logarithmic chirps, respecting the scaling symmetry, would be preferable. The quasi-hyperbolic cutoff solid lines are described by: $\tau \left(f\right)={c}_{i}{t}_{0}{f\left(kHz\right)}^{-b}$, where the coefficients *t*_*0*_ and *b* are positive constants (typically *t*_*0*_ = 10–15 ms and *b* = 0.7–1) derived from measurements of the SFOAE and TEOAE phase gradient delay [18], and the coefficients *c*_*i*_ = (− 0.5, 0.5, 1.5) are used to select the nonlinear distortion component (between *c*_1_ and *c*_2_), the first reflection component (between *c*_2_ and *c*_3_) and the multiple intracochlear reflection components (above *c*_3_). In Figs. 1 and 2, *t*_*0*_ = 12 ms and *b* = 0.8 were used. A single filtering operation allows selecting a specific source across a wide frequency interval. Note that a value of *b* slightly lower than unity is generally necessary, reflecting the slow scaling symmetry breaking associated with the dependence of tuning on frequency.

As anticipated in the introduction, in the intermodulation distortion product OAE (DPOAE) case, a constant ratio between the frequencies of the stimuli is necessary to preserve the scaling symmetry of the experiment, and to be able to predict the frequency dependence of the group delay for the two DPOAE components. Indeed, as the generation region of the 2*f*_1_-*f*_2_ intracochlear distortion product (IDP) is near *x*(*f*_2_), it moves in a scaling symmetric way with the frequency 2*f*_1_-*f*_2_ only if the ratio *f*_2_/*f*_1_ is kept constant, because in this case also the ratio *f*_DP_/*f*_2_ is constant. On the other hand, a paradigm in which the generation place is fixed, as the *f*_2_-fixed paradigm, also permits to some extent to predict the expected frequency dependence of the phase-gradient delay for a scale-invariant cochlea [31, 53].

The optimal representation of the different OAE components provided by the wavelet transform permits effective filtering [54], capable of unmixing OAE components of different physiological meaning and dramatically improving the signal-to-noise ratio (SNR). Providing a visual representation in which these components are separated allows one to optimize both analysis and filtering. Indeed, the optimal compromise between frequency and time resolution, which depends on a single free parameter (the relative bandwidth Δ*f*/*f* of the mother wavelet spectrum), can be easily found by visually inspecting the t-f plots. The same applies to the choice of the width of the hyperbolic filtering regions that allow one to select OAEs associated with a specific generation mechanism and/or relative displacement (with respect to the resonant place), which may also be found using adaptive algorithms [18]. In small mammals, the shorter OAE delay, measured in the number of cycles to use a scaling unit (or the broader relative bandwidth Δ*f*/*f*) may make it difficult to design an effective filter procedure. Emissions from different mechanisms may overlap along the delay dimension, but, as a general rule, the mother wavelet relative bandwidth should be increased to match that of the typical animal response, for improved source unmixing. To some extent, the same filtering purpose may also be achieved without time-frequency representation and analysis, using either IFT time-domain filtering techniques [55] with variable delay windows in different frequency subranges, and/or least square fit (LSF) procedures applied to swept-tone OAE acquisition [52, 56], with different chirp rates and windows length used to optimally select different delay components. The wavelet technique has the advantage, with respect to the IFT option, of performing a single filtering operation over the whole frequency range, and, with respect to the LSF option, of providing a visual representation in the t-f plane of how the filtering regions actually match, or do not match, the source distribution.

The optimal scale-invariant wavelet representation of OAEs also allows one to visually appreciate details of the OAE response that are not visible in the frequency domain and may be embedded in noise in the time and frequency domains. This is shown in Fig. 2, where the weak DPOAE response of a subject affected by Parkinson’s disease is shown in the frequency and time-frequency domain. Although in the frequency domain, the SNR is very low over the whole frequency range, the zero-latency component emerges from the homogeneous noise background in the time-frequency representation. Such data would be typically rejected by a standard statistical analysis based on the SNR, either global or averaged over frequency bands. Obviously, the fraction of rejected data should be minimized, particularly when, as in this case, each session measurement may represent a stress factor for a sensitive patient. In such patients, the usual way of improving the SNR by extending the integration time may be impracticable, because of either excessive stress or lack of stationarity of the response.

Recently, a DPOAE generation mechanism has been proposed [57] involving reflection by roughness associated with the spatial modulation of the strength of the cochlear amplifier. In such a case, a peculiar time-frequency signature of this component had been theoretically predicted [58], i.e., the occurrence of symmetric positive and negative delay components in the delay-frequency plane. The time-frequency representation is optimal to verify this hypothesis and time-frequency filtering may be an effective way to measure the relative weight of such components.

### SFOAE and TEOAE Representation and Filtering

To some extent, similar considerations also apply to the SFOAE and TEOAE representations. These OAEs are mostly generated by a single mechanism, coherent reflection, but a fine structure is observed also in the SFOAE and TEOAE spectra. In this case, for each frequency, there is interference among components generated by the same mechanism in different cochlear regions, and among components associated with multiple intracochlear reflections. These interference phenomena may also add extra complexity to the DPOAE fine structure, which is mostly due to the interference between the components coming from the two different mechanisms. For SFOAEs and TEOAEs, the source distribution is strongly inhomogeneous, and the sources are not point-like. Indeed, for each frequency, the coherent reflection filtering (CRF) mechanism [31, 59] selects different regions of coherent reflection localized within the spatial width of the response peak, yielding reflected wave packets with different group delays and bandwidths. As a consequence, the time-frequency representation of these OAE responses consists of a collection of spots, of given delay and frequency widths [60]. Neglecting intracochlear reflections, different spots at the same frequency correspond to differently spatially localized sources. The possibility of separating in the t-f domain two such sources at the same frequency is related to the intrinsic spatial width of the sources, to the steepness of the delay-position function and to the time resolution of the *t-f* analysis. Indeed, the delay width of a source is related to its spatial width and to the variation of the slope of the BM response phase (group delay) within that region. Near the peak of the BM response, this variation may be large; therefore, regions of small spatial width and close to each other may appear as separate spots of different delay in the t-f plane, whereas more basal sources, generated within regions where the group delay is slowly varying, may appear as a single spot localized along the time axis even if they are associated with a coherent source extended over a wider spatial region. Optimizing the frequency-time resolution balance of the mother wavelet could help, but the overlap between the intrinsic delay widths of the basal spots cannot be overcome. These concepts were formalized in [60] by defining a local reflectivity source.

**Fig. 3:**
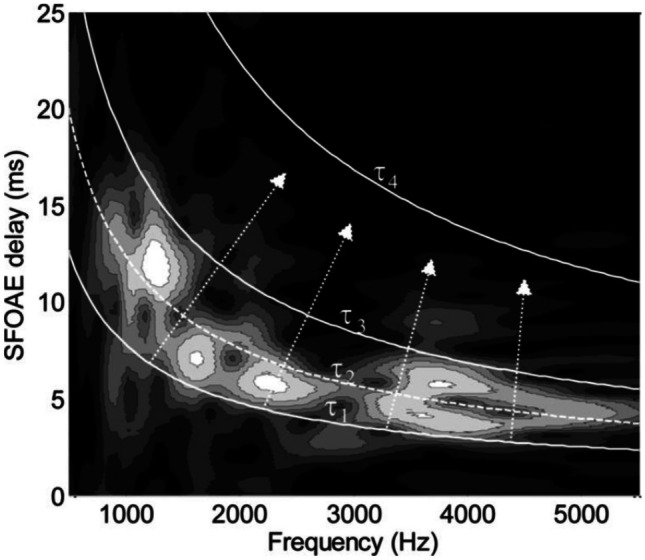
Time-frequency representation of an SFOAE response (from [18]), with hyperbolic curves delimiting across frequency a set of scaling equivalent regions with the same physics (e.g., nonlinear distortion below $\tau_{1}$, absent in the SFOAE case, single reflection from a region slightly basal to the peak (defined here as short-latency, or SL) between $\tau{1}$ and $\tau_{2}$, single reflection from the peak (long-latency, or LL) between $\tau_{2}$ and $\tau_{3}=2\tau_{2}$, double reflection (double-latency, or DL) between $\tau_{3}$ and $\tau_{4}=2\tau_{3}$, multiple reflections above $\tau_{4}$). With respect to Fig. 1, the dotted cutoff line is added, which separates first reflection component coming from different regions (SL and LL) within the peak region, which are separated along the delay dimension because in that region the phase gradient delay is rapidly varying with the source position

Localization of the OAE source of a given frequency must be interpreted in a scale-invariant way, as relative to the peak of the BM response of that frequency, taking into account the finite-width spot-like nature of the contributions to the OAE response from coherent reflection. The t-f representation shows the distribution of the sources in this scale-invariant way, with sources sharing the same spatial shift relative to the best place of that frequency distributed along hyperbolic lines in the t-f plane (see Fig. 3). In this representation, the distribution of the OAE sources along the scale-invariant coordinates (same physics at different frequencies moving along the hyperbolas, different physical phenomena moving orthogonal to them) is immediately visible.

Sisto et al. [18] applied the wavelet filtering procedure described in Fig. 3 to TEOAE and SFOAE spectra from the same ear, showing that the first-reflection components coming from more basal sub-regions (SL, below the dashed line in Fig. 3) have systematically steeper I/O functions than the LL components, consistently with a less compressive behavior of the BM response basal to the resonant peak. Double reflections show a strongly compressive behavior, because the cochlear nonlinear gain is experienced twice during their TW path.

**Fig. 4:**
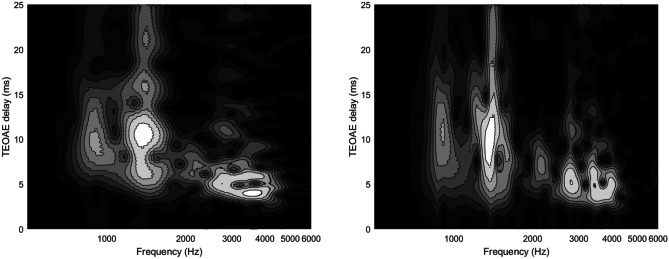
SSOAE and multiple reflections in two wavelet representations of the same TEOAE response to a 60 dB click stimulus. In the right panel, the intrinsic frequency resolution of the analysis in improved by a factor of two, and correspondingly, the time resolution is worsened by the same factor. Logarithmic units are used for the frequency axis to show the invariance across frequencies of the intrinsic relative frequency resolution Δ*f*/*f*

In time-frequency representations, SSOAEs emerge from the hyperbolic SFOAE (or TEOAE) pattern as an amplitude-modulated vertical bright line, with an envelope of decaying amplitude in the first tens of ms, eventually reaching a stationary amplitude for long delays, as shown in [18]. In Fig. 4, such a behavior is shown using a log scale for the frequency axis, for two different choices of the trade-off between time and frequency resolution of the wavelet analysis. The modulation period corresponds to the round-trip delay between each intra-cochlear reflection, confirming the interpretation [61] of SSOAEs as due to the concurrence of coherent (in a standing-wave sense) intracochlear reflections and round-trip gain larger than unity (in the long-delay, low-amplitude limit). On the other hand, in the case of narrow-band SOAEs, other t-f methods may be more effective than the wavelet transform, as previously mentioned.

Another useful feature of the t-f OAE representation is related to the detection of artifacts. Jedrzejczak et al. [62] proposed using the localization of the ringing artifact within a specific region of the t-f plane to filter TEOAEs recorded with a linear acquisition paradigm. The SFOAE residuals yielded by the suppression or compression method [63] may be “contaminated” by artifacts associated with transitory changes of the probe stimulus level effectively reaching the cochlea. If slow chirps lasting several seconds are used for the probe and suppressor stimuli, a fluctuation of the middle ear transmission during one of the probe chirps due, e.g., to swallowing, would cause a spurious zero-delay residual contribution localized along the frequency axis over a frequency interval corresponding to the perturbed chirp fraction. As the probe level is typically much higher than the SFOAE residual, and as the differential SFOAE acquisition paradigms (both compression and suppression) are based on linear differences, even a small fraction of the probe amplitude would give a significant contribution to the average residual. Such spurious contributions could be easily identified in the t-f domain from their null group delay, so coupling a nonlinear acquisition method to time-frequency filtering in the first-reflection hyperbolic band would reject both noise and such artifacts without rejecting the good data coming from the time fractions of the chirp that were not perturbed. Again, the t-f representation and filtering help improve the reliability and the SNR of the OAE data.

### OAE Group Delay, Cochlear Tuning, and Stimulus Level

Contrary to expectations based on simplistic active filter interpretations of the OHC amplification, the group delay of OAEs generated by coherent reflection does not vary as rapidly with stimulus level as does the gain of the response [64]. In other words, the inverse proportionality relation between response amplitude and bandwidth that is typical of a resonant passive filter is not observed in the OAE response. Moreover, an accurate time-frequency analysis of the SFOAE and TEOAE response at different stimulus levels shows that elementary components (wave-packets or “spots” in the t-f domain) of almost constant delay are present in the response and that increasing the stimulus level only increases the relative weight of the more basal sources, associated with shorter delay spots [18]. Indeed, CRF components are localized at a specific place, and the relative intensity of the corresponding spots depends on the shape and width of the BM resonant peak. As a consequence, the average delay of the first reflection component (obtained by filtering within a hyperbolic band that excludes multiple delay spots associated with multiple intracochlear reflections) may be used as a stable measure of cochlear tuning [65]. The objective estimate of tuning is an important application of OAE research [3, 64, 66], which is particularly sensitive to noise and to systematic errors related to the interference between sources of different group delay, including the multiple intracochlear reflections that in some cases (when the round-trip gain equal to unity condition is reached for a level above the noise floor) are classified as SSOAEs. The t-f representation and filtering option is generally necessary to improve the quality and stability of OAE-based estimates of tuning [54, 65, 67]. In particular, the delay-frequency function of each OAE component may be more effectively computed as the average of the delay $\tau$ within the corresponding filtering band, weighted, for each frequency, by the wavelet coefficient *WT*_*x*_($\omega , \tau$) squared. This procedure yields a much more stable estimate of the cochlear delay (and tuning), with respect to taking the derivative of the phase-frequency function.

The fact that group delay is a slowly varying function of the stimulus level has also the important consequence of permitting the same choice at all stimulus levels of the t-f hyperbolic filtering regions used for unmixing distortion components, as well as first- and multiple-reflection components, as shown for SFOAEs and TEOAEs in [18]. The individual variation of the OAE group delay among adult subjects of different ages is also remarkably small [68], allowing one to use the same filtering regions also in cross-section studies involving large populations.

## Conclusions

The scale-invariant wavelet time-frequency representation is particularly suitable to analyze OAEs, due to the scaling symmetry of the cochlea and to the conformity (unconformity) to this symmetry of the two main OAE generation mechanism, nonlinear distortion, and coherent reflection from cochlear roughness. Several examples have been provided in this report that highlight some obvious advantages of the wavelet analysis in terms of immediate visualization of the relevant physical properties of the response and of the design of effective filtering procedures. The time-frequency filtering is capable of improving the specificity (by unmixing OAE components of different physiological meaning, generated by different mechanisms and/or at different places) and the sensitivity (by effectively removing most of the noise while including the whole desired signal component) of OAE-based diagnostic techniques.
